# High-normal blood glucose levels may be associated with decreased spatial perception in young healthy adults

**DOI:** 10.1371/journal.pone.0199051

**Published:** 2018-06-14

**Authors:** Rima Abdul Razzak, Abdulla Faisal Alshaiji, Abdulrahman Ahmed Qareeballa, Mohamed Wael Mohamed, Jeff Bagust, Sharon Docherty

**Affiliations:** 1 Department of Physiology, College of Medicine & Medical Sciences, Arabian Gulf University, Manama, Bahrain; 2 College of Medicine & Medical Sciences, Arabian Gulf University, Manama, Bahrain; 3 Faculty of Health and Social Sciences, Bournemouth University, Fern Barrow, Poole, Dorset, United Kingdom; 4 School of Health and Social Care, Bournemouth University, Fern Barrow, Poole, Dorset, United Kingdom; Weill Cornell Medical College Qatar, QATAR

## Abstract

The negative effects of high normal glucose on cognitive function were previously reported in euglycemic individuals of middle age and the elderly population. This study aimed at examining the effect of baseline blood glucose levels on spatial ability, specifically verticality perception on the computerized rod and frame test (CRFT) in young healthy adults. 63 healthy male medical students (age range from 18–23 years), of whom 30 were non-fasting outside the month of Ramadan and 33 fasting during Ramadan of the year 2016, were recruited in order to create varying degrees of glycemia during which verticality perception was carried out. Baseline blood glucose reading was obtained prior to commencing the CRFT test. Blood glucose levels at the time of testing decreased as the duration between the last meal and testing increased. A blood glucose range of 62–117 mg/dl was achieved among participants for this study. Linear regression analysis showed that blood glucose level at testing correlated positively with all alignment spatial error parameters, indicating a probable reduction of spatial perception ability with higher blood glucose levels. These results are consistent with other cognitive studies in older healthy humans and emphasize the critical impact of early glucose dys-homeostasis on cognitive function. They also indicate that elevated blood glucose may affect cognitive functioning outside of the usual complications of diabetes.

## Introduction

The human brain utilizes glucose as its sole fuel except during prolonged starvation and lacks fuel stores, rendering a continuous supply of glucose essential for its normal function and survival. Specially during complex cognitive processing, demand for glucose is high, and glucose/energy consumption is increased in parallel with the increased neural activity [1–3]. Paradoxically, hyperglycemia can have detrimental effects on cognition, with its most common manifestations of cognitive deficit being neural slowing, attention deficit, executive functioning, and learning and memory [4]. Such effects are profound and well established in people with Type 1 and Type 2 diabetes, with high glycated hemoglobin (HbA_1c_) levels predicting cognitive deficit and its development over time [5, 6] in such patients.

The negative effects of hyperglycemia on cognitive function can manifest outside of the usual complications of diabetes and extend to euglycemic individuals mainly of middle age and elderly population [7]. Higher average daily glucose levels have been related to an increased risk of dementia in the elderly [7, 8] and to poorer performance on tests that measure delayed recall, learning ability, and memory consolidation [9]. High normal glycemic indices were also associated with adverse changes of some brain regions in cognitively healthy individuals, such as reduction of volume and microstructure [10–12].

Although most focus has been on older euglycemic individuals, the effect of high normal blood glucose levels on cognitive function in euglycemic healthy young adults is also worth exploring. This study aims to assess in healthy young adults the cognitive ability of visual vertical (SVV) perception. SVV is a measure of the accuracy by which individuals internally represent their position in space relative to gravity. Such internal estimates of the direction of gravity are continuously updated because of frequent changes in head and trunk positions, [13], and as such SVV perception is a very important factor for spatial/gravitational orientation and the maintenance of posture and equilibrium [14]. Our interest in this cognitive ability relates to findings of a previous study investigating the effect of glycemic control on visual vertical (SVV) perception in elderly asymptomatic (without peripheral neuropathy or retinopathy) type 2 diabetic patients. Findings of that study indicated to decline in spatial perception ability during visual conflict in comparison to age-matched healthy controls, suggesting subclinical impairments of spatial orientation in patients with diabetes [15]. However, verticality perception in diabetics was not predicted by blood glucose levels or long term glycemic control (HbA_1c_ levels) in that study.

Establishing whether high normal blood glucose levels in healthy young adults can have adverse effects on spatial (SVV) perception is worthy, considering that maintaining an average normal glucose level throughout the day may be an important part in performing more efficiently and effectively in activities that require optimal spatial orientation, such as navigation [16], sports [17, 18] and driving [19, 20].

## Materials and methods

### Subjects

63 healthy male medical students (age range from 18–23 years) were recruited for this study at the College of Medicine and Medical Sciences (CMMS) at the Arabian Gulf University (AGU) in Bahrain. Of these 30 were non-fasting outside the month of Ramadan and 33 fasting during Ramadan of the year 2016 (June 6—July 5). The duration of fast from dawn (3:12–3:18 am) to dusk (6:29–6:35 pm) ranged from 15 hours, 18 minutes on the first day to 15 hours, 2 minutes on the last day of Ramadan month.

The participants in the fasting group were recruited at different stages of Ramadan, 19 of which were assessed at the end of the first week of Ramadan. 14 other subjects were tested at the end of the 3^rd^ week of Ramadan in order to reduce any testing bias encountered at the beginning of Ramadan in which the body has not fully adapted to changes in feeding habits, sleep cycles [21, 22] and other circadian rhythms [23, 24].

All participants were asked to provide information about the night before their cognitive testing, such as time of their last meal and the number of hours of nocturnal sleep they had that night, as well as quantity of their sleep in general. At the beginning of the test, blood glucose levels were measured with the ACCU-CHEK ACTIVE (Roche, Germany) glucometer. All the subjects gave written informed consent, and the study was approved by the Research and Ethics committee at AGU.

### The computerized rod-and-frame test (CRFT)

The test is a modified version of the computer Rod and Frame test [25]. This clinical exam evaluates a subject’s ability to position a line to vertical position without a vertical reference, with most people normally deviating within ±2° from gravitational vertical [26–28]. The ability to do so depends on the integration of visual and mainly vestibular otolithic inputs centrally in the brain [14]. The CRFT program incorporates two different but related tests; SVV in which the rod is displayed on a blank background and measures the participant’s internal representation of vertical; and the Rod and Frame in which the rod is rotated within a tilted frame to determine how a distracting background influences the perception of vertical [29, 30].

**Fig 1:**
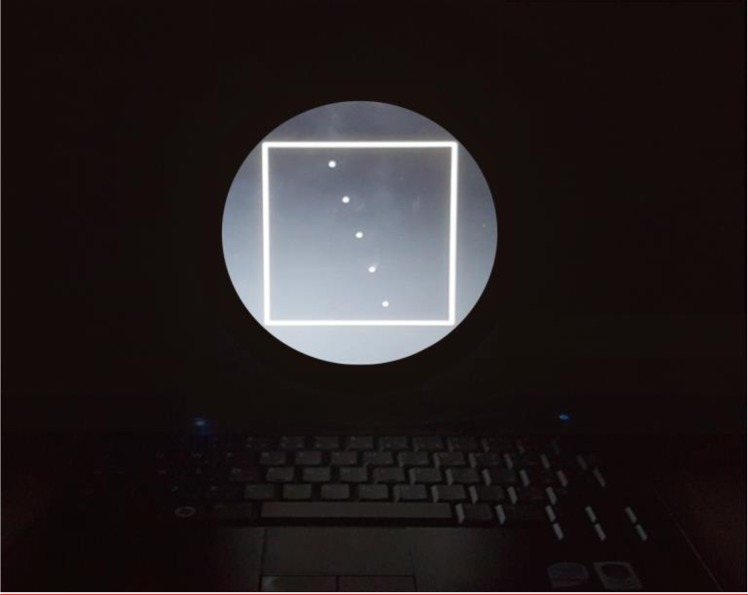
Computer set up for SVV recording. Concealment of the vertical edges of the laptop to obscure from participants any cues of verticality in the laptop frame.

The rod consisted of five white dots displayed in the center of the screen and had two starting positions, tilted 20 degrees in either a clockwise or counter clockwise direction from gravitational vertical. A round black paper ring was stuck on the laptop screen to conceal its edges and reduce clues to verticality, while exposing the rod and frame presentation in the center of the screen (Fig 1). The test was performed in a dark room minimizing further any vertical cues within the room.

**Fig 2:**
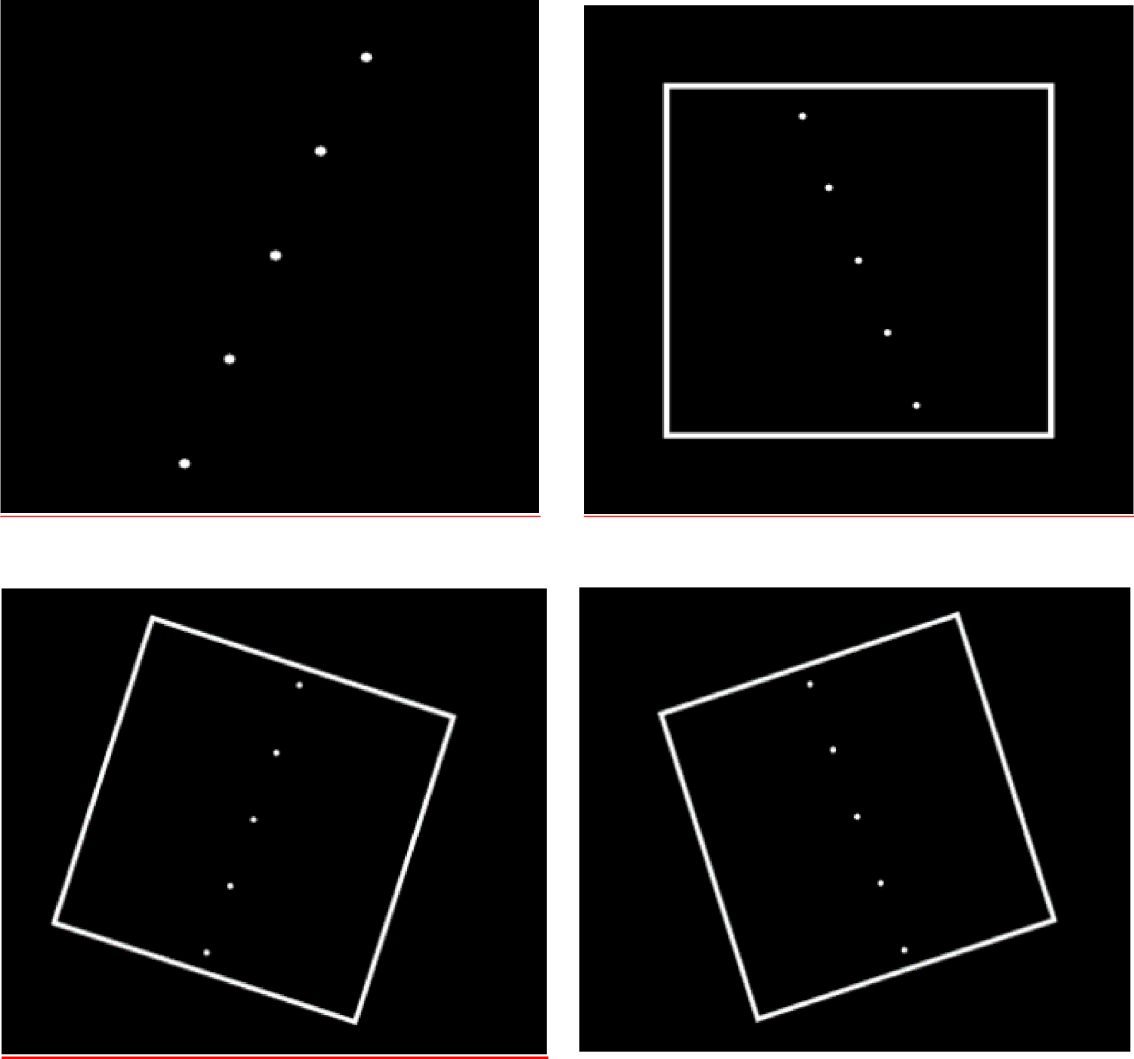
Presentations of “rod” and frame during testing. The order of presentation was randomly assigned by the computer.

The vertical test comprised 18 presentations, the first two of which were for instruction purposes and were not included in the analysis. The remaining 16 presentations consisted of four replicates where the frame, a white square presented on a homogenous black background (Fig 2), was either (i) absent (SVV; No frame); (ii) un-tilted (frame^0°^); (iii) tilted 18 degrees in a clockwise direction (frame^+18°^); or (iv) 18 degrees in a counter-clockwise direction (frame^-18°^). The order of presentation of these permutations of frame and dots was assigned by the computer from 4 randomized sequences.

### Procedure

To examine the effect of baseline blood glucose levels on verticality perception, a blood glucose reading was obtained prior to commencing the CRFT test. Participants were seated in front of the laptop screen and asked to look straight at the screen keeping their head in a fixed position without tilting or turning. They were instructed to rotate the dots using the right and left mouse buttons to a position perceived to be vertical. The dots rotated around their virtual midpoint in 0.5 degree increments. When the participant was satisfied with the alignment of the dots, the program was advanced using the space bar of the computer keyboard. Positioning errors were recorded by the computer in degrees from gravitational vertical and were only accessible at the end of the recording session. There was no time limit for individual adjustments.

#### Spatial error calculations

Deviations from vertical were recorded in degrees as positive values if the deviation was in a clockwise direction, and negative values for counter-clockwise deviations. These values were imported into Excel 2003 and used to calculate the mean signed errors and the mean absolute (unsigned) errors for the four frame conditions (n = 4 in each case) for each participant. For both non-fasting and fasting participants, paired t-test analysis showed that there were no significant differences between the absolute errors produced for the tilted frame^+18°^ and tilted frame^-18°^ (Non-fasting: t = 0.39, *P* = 0.70; Fasting: t = 0.78, *P* = 0.44), allowing the mean absolute errors to be combined to form a mean tilted frame score denoted by Mean^18°^.

The effect of the tilted frame on rod alignment was measured by calculating the spatial frame effect (SFE) [31, 32] by subtracting the mean signed error recorded in the presence of the un-tilted frame from the mean signed error recorded in the presence of a tilted frame (SFE^-18^ or SFE^+18^). This provided a measure of the influence of the surrounding tilted frame on the perception of vertical. The asymmetry of the errors induced by the tilted frame around the un-tilted frame error was investigated by deriving an asymmetry index (δ°) from the signed data. For each individual participant this was calculated by summing the differences between the mean signed errors in the tilted frame conditions (Frame+18, Frame−18) and the mean signed error in the Frame 0 condition.

$$Asymmetry\;Index\;(\delta )=({Frame}^{18^{{}^{\circ}}}-\;{Frame}^{0^{{}^{\circ}}}\;)+({Frame}^{-18^{{}^{\circ}}}-\;{Frame}^{0^{{}^{\circ}}}\;)$$

A symmetrical response to the frame tilt is indicated by a value of δ close to 0. Larger values indicate increased asymmetry, with the sign showing the direction of the skew. The direction of the skew is important for each individual, but when assessing group data there is a risk of positive and negative values canceling, and so the absolute value of δ, |δ°|, was used in the analysis of the results.

#### Statistical analysis

All statistical analyses were carried out using INSTAT (GraphPad Inc.). Data were tested for normality using the Kolmogorov-Smirnoff method. Data was reported as mean ± SD, and comparison of means between non-fasting and fasting participants was done with the unpaired t-test. Pearson’s correlation and linear regression tests were performed to examine associations between baseline glucose level and other parameters. Level of significance was set at *P* < 0.05.

## Results

### Blood glucose levels

All data in Table 1 was normally distributed. The time without food for the non-fasting participants ranged from 10 minutes to 6 hours, and that for the fasting participants was between 7 and 15 hours. Out of the 30 non-fasting participants, 7 (23%) were tested within 1 hour and 8 (27%) within 2 hours after their last meal.

**Table 1:** Measures of glucose and CRFT unsigned spatial errors under different frame conditions.

	Non-Fasting (n = 30)	Fasting (n = 33)	t (*P*)
**Blood glucose level (mg/dl)**	85.53 ± 13.26 (62.0–117.0)	80.61 ± 9.94 (63.0–102.0)	1.69 (*0*.*096*)
**SVV**^**°**^	0.80 ± 0.37 (0.25–2.00)	0.60 ± 0.35 (0.12–1.75)	2.21 (*0*.*03*)
**Mean**^**18°**^	1.26 ± 0.54 (0.69–3.44)	1.14 ± 0.50 (0.44–2.06)	0.96 (*0*.*34*)
**|δ**^**°**^**|**	1.24 ± 0.95 (0.25–4.87)	1.04 ± 0.99 (0.11–4.63)	0.81 (*0*.*43*)

Values as mean ± SD and range. |

δ°| is the absoulte asymmetry of errors during tilted frame conditions. Unpaired t-test and *P* significance level.

**Fig 3:**
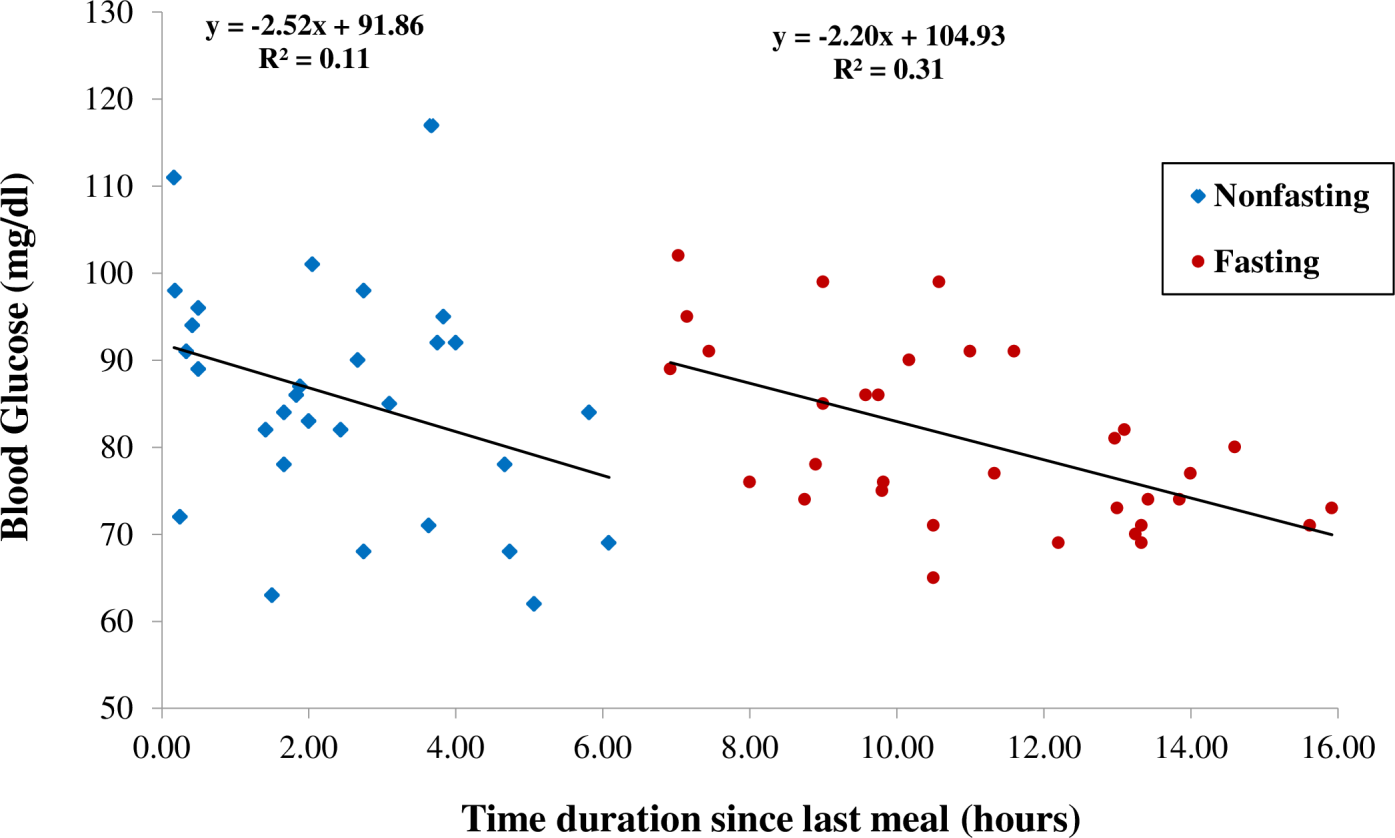
Duration of food omission and glucose levels. Effect of time spent without food on blood glucose level in both non-fasting (n = 30) and Ramadan fasting (n = 33) participants.

Table 1 shows the participants’ mean blood glucose level at the time of testing. Difference in variance for glucose levels between the non-fasting and fasting participants was not significant (Difference between SDs: F = 1.781, *P* = 0.06), and unpaired t-test analysis reveals no significant difference in mean glucose values between the two groups. Fig 3 shows that for the non-fasting group, there was an almost significant correlation between the time duration without food and the level of blood glucose (r = -0.33, *P* = 0.08; n = 30). For the fasting group, blood glucose levels dropped significantly (r = -0.56, *P* = 0.0007; n = 33) with increasing fasting duration at the time of testing. The blood glucose levels for the combined cohort did not drop beyond 62 mg/dl.

### Unsigned spatial errors

The reported spatial errors on the CRFT are the absolute (unsigned) values. All data related to alignment error followed a Gaussian distribution as tested by the Kolmogorov and Smirnov method. The mean values for the unsigned alignment errors shown in Table 1 fall within the normal range for all frame conditions. Paired t-test analysis for each group revealed that inclusion of a tilted frame significantly increased the alignment error in comparison to the absent reference frame condition, with a mean difference of 0.47° in the non-fasting group and 0.53° in the fasting group (Non-Fasting: t = 5.26, *P* < 0.0001; Fasting: t = 9.26, *P* < 0.0001).

Comparsion analysis between the two groups of participants shows that the only significant difference between them was in their perception of vertical in the frameless presentation (SVV) Table 1. As the difference between the two groups in SVV perception is less than the precision of the system of 0.5° and is less than one mouse click difference, any functional differences can be excluded. Accordingly we pooled all participants with a cohort of 63 participants yielding a blood glucose range of 62–117 mg/dl and mean of 83.47 ± 12.78 mg/dl. For the combined cohort, blood glucose level at testing decreased significantly with longer non-feeding intervals (r = -0.38, *P* = 0.002; n = 63).

#### Blood glucose level and spatial verticality perception

**Fig 4:**
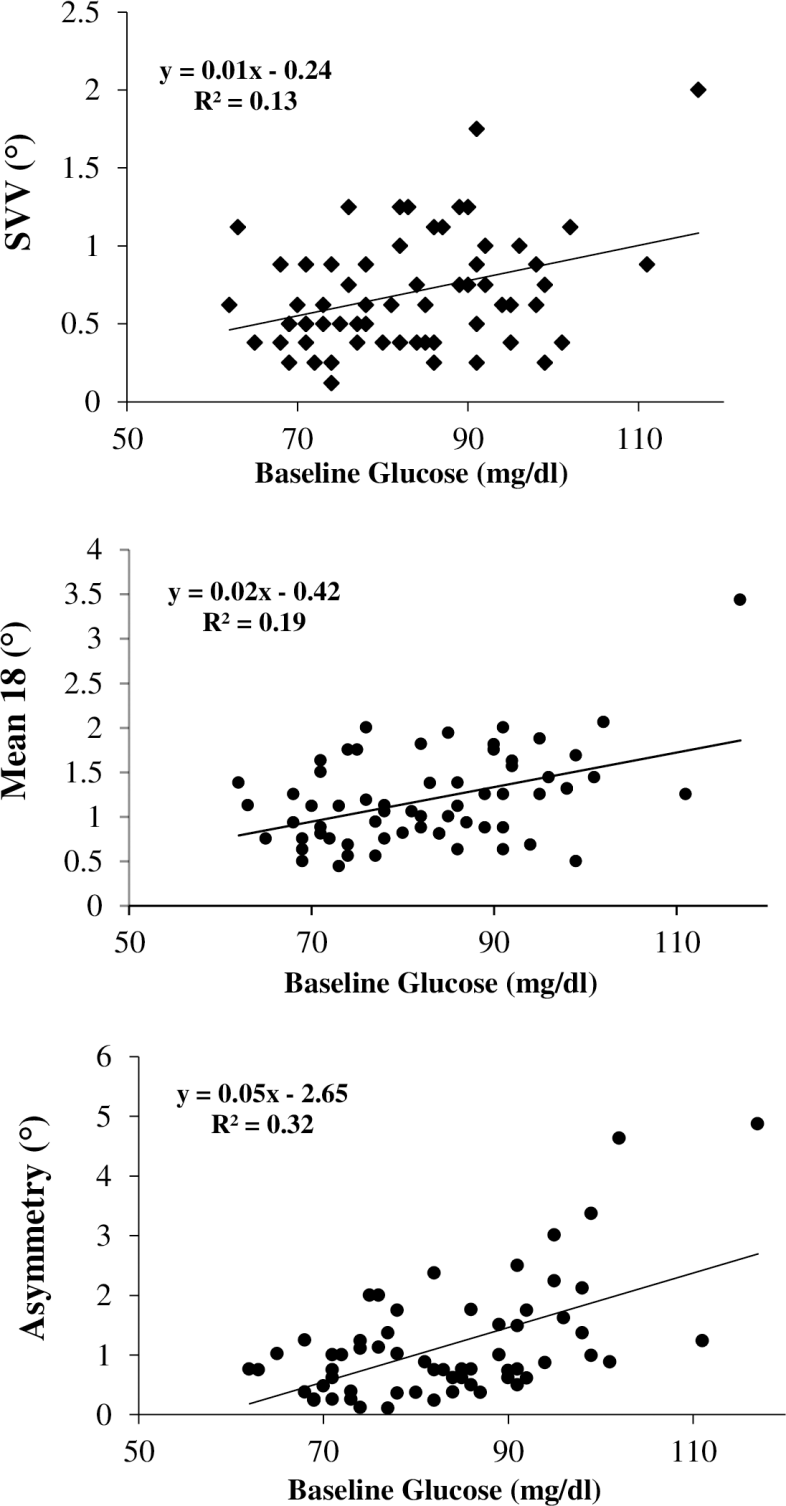
Blood glucose levels and verticality perception. Relationship between baseline glucose level and unsigned spatial measures of verticality on CRFT in non-fasting and Ramadan fasting participants pooled together (n = 63).

For the combined cohort (n = 63), SVV values ranged between 0.12 to 2.00 degrees. Mean 18 alignment error values and the asymmetry index *|δ*^*°*^*|* ranged from 0.44 to 3.44 and 0.11 to 4.87 respectively. Linear regression analysis showed that blood glucose level at testing correlated positively with alignment errors in both frame conditions (SVV: r = 0.36, *P* = 0.004; Mean 18: r = 0.44, *P* = 0.0003) and with the derived asymmetry index (*|*δ^*°*^*|*: r = 0.56, *P* < 0.0001) (Fig 4).

#### Fasting and sleep duration and verticality perception

For the combined cohort, the fasting duration at testing ranged from 0.17 to 15.9 hours with a mean fasting duration of 7.26 ± 4.89 hours. Sleep duration during the night before testing ranged from 0.00 to 12.0 hours, with a mean sleeping time of 5.90 ± 2.21 hours for the combined cohort. Most participants however explained that these sleep durations were not consistent. Standard multiple regression analyses showed that our models, which included the independent variables fasting duration and sleep duration explain 12.38% of the variance in SVV errors (*P* = 0.02), 5.24% of the variance in Mean^18°^ error (*P* = 0.20) and 8.50% of the variance in asymmetry index (*P* = 0.07). Of those two independent variables, only fasting duration made a statistically significant contribution (t-ratio = 2.87, *P* = 0.006) to SVV variance.

## Discussion

This is an important study as it is one of a few that address the effect of high- normal glucose levels on one cognitive ability in young healthy adults. Our study shows a negative correlation between blood glucose levels, within the normal range, and a cognitive task assessing verticality perception, which is important for spatial orientation. Most studies on the effect of blood glucose on cognitive function have considered mainly the influence of a glucose containing drink on cognitive tests, and others utilized insulin clamps to create varying bands of glycemia during which cognitive testing is carried out. However, in our study we have utilized a wide range of normal blood glucose levels for cognitive testing. To ensure glucose levels were in the low normal range, we recruited healthy individuals observing Ramadan fasting during which fasting from dawn until breaking fast at sunset lasted more than 12 hours. This provided an array of blood glucose levels on the low end of the physiological range, depending on the time of the last meal ingested and the time of cognitive testing. We also recruited outside of the month of Ramadan participants under normal feeding conditions with different durations between their daytime meals, and different glucose levels.

It is evident that longer non-feeding durations produced lower blood glucose levels at the time of testing in both fasting and non-fasting participants. Fasting participants who chose to omit their Sahour meal at dawn provided long fasting durations at the time of testing, sometimes exceeding 15 hours especially when testing was carried out later in the fasting day. But because Ramadan fasting is intermittent and not prolonged, hypoglycemic excursions were prevented, and fasting glucose levels rarely fell below the acceptable physiological range of 65–70 mg/dl [33]. Even outside fasting, some of the non-fasting participants extended the duration between daytime meals to many hours, also creating blood glucose levels at the lower end of the normal range at time of testing, with the lowest value for blood glucose level in our study being 62 mg/dl in a non-fasting participant.

The challenge of studying a wider range of normal glucose levels does not lie in the lower end of the range, as this was consistently observed in participants who fasted for long hours and occasionally in non-fasting subjects who skipped meals. Our challenge was with increasing the upper end of the range, and this is reflected by our highest glucose level of only 117 mg/dl in this study. Attaining higher glucose levels would have involved testing non-fasting participants within two hours after their last meal, because in such a time, blood glucose levels typically rise for half an hour, after which they fall, returning to near baseline levels [34]. Although 50% of our non-fasting participants were tested during this two-hour duration, the highest glucose level obtained was 111 mg/dl ten minutes after the last meal. Even with experimental elevation of blood glucose levels in which healthy participants fast overnight and then undergo cognitive testing within two hours from drinking glucose drink, it is not guaranteed that we would have consistently obtained high glucose levels for two reasons. First, participants may differ in their ability to regulate blood glucose and the rate at which their blood glucose levels return to baseline, and secondly, the maximum level usually obtained in healthy participants with such a manipulation is 140 mg/dl [34].

The results in our study indicate that in young healthy adults, higher glucose levels in the normal range are not necessarily free of adverse effects on the CRFT, as higher blood glucose levels were positively correlated with larger alignment errors and asymmetries. According to Cohen’s conventions [35], the effect size of glucose level on alignment errors was moderate in both frame conditions and large on the derived asymmetry index, however glucose levels in this study cannot predict, but may have some influence on verticality perception. These results are resonant to our previous study in which blood glucose levels in patients with diabetes portrayed a hyperglycemic range [15] and had a higher rate of larger alignment errors during visual conflict in comparison to euglycemic controls. For alignment errors, 26% of the diabetic participants exceeded a calculated top limit of 3.3 degrees from control group data, and almost 13% exceeded the maximal allowed value of 4 degrees. Even though the values of the alignment errors in our study are still within the acceptable range [26–28, 36, 37], the larger errors in participants with glucose levels at the high end of the normal range may serve as early projections of future spatial perceptual dysfunction if glucose levels progressively increased to the stage of inadequate glucoregulation. Further investigating of such an indication should be considered.

The above findings on verticality perception may seem equivocal, as it is intuitive to assume that brain areas involved in SVV processing could benefit from higher normal glucose levels, considering that there is increased neural activity during SVV judgement and alignment tasks in brain areas representing the neural basis of spatial reference frames [38]. Our results however concur with other studies in older healthy people, in which a negative effect of high normal glucose levels on cognitive function has been referred to. Specifically, these studies have reported that daily glucose levels even within the range of 90 to 120 mg/dl increased risk of dementia in the elderly [7, 8], while others related associations of high fasting HbA_1c_ levels with reduced hippocampal volume and microstructure [10], high normal fasting plasma levels with hippocampal and amygdalar atrophy [11], and high normal glucose levels with decreased frontal cortices volume in older healthy individuals [12].

The underlying pathophysiological mechanisms of how blood glucose could be associated with cognitive deficits still remain to be elucidated in euglycemic individuals with high normal blood glucose levels, just as in diabetics. Some speculations about how elevated blood glucose could be associated with cognitive dysfunction include a direct toxic effect of glucose on the brain, association of glucose with inflammatory processes and defects in brain insulin signaling, or other processes that may have a detrimental effect on the brain [7].

It is also established that some cognitive functions such as psychomotor and cognitive speed, vigilance and executive attention, working memory, and cognitive abilities can be affected by sleep loss [39]. In our study, the duration of sleep the night before cognitive testing ranged from no sleeping to 12 hours of sleep but had no effect on any parameter of verticality perception. This could be due to the non-chronic sleep restriction for our participants who slept for short hours the night before testing, or because individuals differ in the degree of their cognitive vulnerability to sleep loss [39].

## Conclusions

This study had some limitations. Relying on one glucose reading that is measuring short-term glucose levels, and on one-time cognitive assessment of participants may not offer conclusive findings. It may have been preferable to include serial glucose measurements and corresponding CRFT testing sessions, however this was not carried out due to the vulnerability of the RFT to learning and practice effects, as alignment errors can be significantly decreased by only one retest [40]. Additionally, this study does not establish a cause and effect relationship between blood glucose levels and alignment errors nor finds any particular “threshold range” of blood glucose level for optimal verticality perception. Despite of these limitations, our results are in line with other cognitive studies in older healthy individuals, and young adults should be aware that even outside of the usual complications of diabetes, daily high normal-range glucose levels may induce temporary cognitive impairment. Accordingly, future studies should be conducted to investigate whether short-term improvement of glucose may maintain or improve verticality perception and daily activities that require spatial orientation such as navigation, sports and driving.

## Supporting information

S1 File Data relevant to the study. The spreadsheet file includes data concerning our non-fasting and fasting participants, blood glucose levels and verticality testing results.
(XLSX)
